# Retraction: FoxD2‐AS1 promotes glioma progression by regulating miR‐185‐ 5P/HMGA2 axis and PI3K/AKT signaling pathway

**DOI:** 10.18632/aging.204664

**Published:** 2023-04-14

**Authors:** Wei Ni, Yaoxiong Xia, Yuxu Bi, Fan Wen, Dong Hu, Lin Luo

**Affiliations:** 1 Department of Neurosurgery, The Third Affiliated Hospital of Kunming Medical University, Kunming 650118, Yunan Province, China; 2 Department of Neurosurgery, Yunnan Cancer Hospital, Kunming 650118, Yunan Province, China; 3 Department of Neurosurgery, Yunnan Cancer Center, Kunming 650118, Yunan Province, China; 4 Department of Radiation Oncology, The Third Affiliated Hospital of Kunming Medical University, Kunming 650118, Yunan Province, China; 5 Department of Radiation Oncology, Yunnan Cancer Hospital, Kunming 650118, Yunan Province, China; 6 Department of Radiation Oncology, Yunnan Cancer Center, Kunming 650118, Yunan Province, China

**Keywords:** lncRNA, FOXD2‐AS1, glioma, proliferation, metastasis

**This article has been retracted:** Aging has completed its investigation of this paper. We found multiple internal duplications and overlap between some of the transwell assay images used for **Figures 3C,E** and **5C,D,F** and data published the preceding year by other authors [1]. The authors reply that “There are some irreproducible data (Figure 2 and 3) in our manuscript.” An institutional investigation has concluded that the figures are unreliable and that much of the additional data in the paper cannot be reliably verified from records. Together, these issues decrease confidence in the integrity of the experimental findings reported. Consequently, all authors agreed that the article should be retracted. The authors sincerely apologize to the scientific community for any confusion and any unintended harm derived from the publication of this paper.

## References

[1] Zhou K, Zhang C, Yao H, Zhang X, Zhou Y, Che Y, Huang Y. Knockdown of long non-coding RNA NEAT1 inhibits glioma cell migration and invasion via modulation of SOX2 targeted by miR-132. Mol Cancer. 2018; 17:105. 10.1186/s12943-018-0849-2 30053878 PMC6064054

